# Association of the visceral adiposity index with femur bone mineral density and osteoporosis among the U.S. older adults from NHANES 2005–2020: a cross-sectional study

**DOI:** 10.3389/fendo.2023.1231527

**Published:** 2023-11-02

**Authors:** Aochuan Sun, Jiayu Hu, Shushangzhi Wang, Fen Yin, Zhengtang Liu

**Affiliations:** ^1^ Graduate School, Beijing University of Chinese Medicine, Beijing, China; ^2^ Xiyuan Hospital, China Academy of Chinese Medical Sciences, Beijing, China

**Keywords:** visceral adiposity index, femur bone mineral density, osteoporosis, U-shaped, cross-sectional study, National Health and Nutrition Examination Survey

## Abstract

**Background:**

The visceral adiposity index (VAI) is a marker of abdominal fat distribution and adipose tissue function. However, the association between VAI and femur bone mineral density (BMD) and osteoporosis is unclear among the U.S. older adults.

**Methods:**

Cross-sectional data for adults aged 60 years and older from the 2007–2020 National Health and Nutrition Examination Survey (NHANES) were included. Multivariable linear and logistic regression were used to evaluate the association between VAI and femur BMD and osteoporosis. We used the smooth curve fitting to address nonlinearity. Moreover, a two-piecewise linear regression model was used to explain the nonlinearity further.

**Results:**

The findings of the multivariable logistic regression models showed that as the VAI value increased by one unit, the prevalence of osteoporosis decreased by 1.2% after adjusting for covariates associated with osteoporosis. The multivariable linear regression models demonstrated that VAI was positively correlated with femur BMD. Further analysis revealed an inverted L-shaped and inverted U-shaped relationship between VAI and femur BMD at different sites.

**Conclusions:**

Our findings indicated that an increased VAI is independently linked to a higher prevalence of osteoporosis among the U.S. older adults. Further analysis reveals that once VAI reaches a certain threshold, femur BMD no longer increases and may even decrease. This suggests that a moderate accumulation of visceral fat may be beneficial for bone health, while excessive visceral fat could potentially have detrimental effects.

## Introduction

Osteoporosis is a group of metabolic bone diseases characterized by reduced bone density, degradation of bone tissue microarchitecture, and an imbalance in bone homeostasis. This condition poses a risk of fragility fractures, which can increase mortality rates among patients. Bone mineral density (BMD) is a widely used metric for assessing bone health (1). Elderly individuals are at a higher risk of developing osteoporosis due to increased disruption of bone synthesis homeostasis with age (2). Femur BMD is a highly effective indicator for the detection and diagnosis of osteoporosis, with a strong correlation to all-cause mortality and mortality from other causes in patients with this condition (3). Especially for older people, hip fractures can be devastating (4). Therefore, it is of great significance to identify the risk factors related to low femur BMD and osteoporosis in elderly people.

Obesity has a complex impact on the development of osteoporosis, with both positive and negative effects on skeletal homeostasis. Several studies have identified a threshold effect of adiposity on BMD based on body mass index (BMI) (5, 6). The percentage of body fat has also been observed to influence BMD. This may be due to abnormal glycolipid metabolism triggered by obesity that affects bone strength (7). Additionally, a moderate amount of fat can contribute to bone protection, and body weight can stimulate bone growth via mechanical stress (8).

In recent years, the visceral adiposity index (VAI), calculated by combining anthropometric measurements and blood biomarkers, has emerged as a novel indicator for assessing obesity. It takes into account various factors such as BMI, waist circumference, triglycerides, and HDL cholesterol to assess the amount of visceral fat accumulation. This compensates for the limitations of BMI and waist circumference in differentiating between muscle and fat content. Some studies have suggested that VAI is superior to traditional BMI or waist circumference in predicting the risk of cardiometabolic disease (9, 10). Moreover, it has shown promising results in predicting and evaluating the risk of diabetes, metabolic syndrome, blood pressure, and endometrial cancer development (11–14). Previous research has indicated that individuals with osteopenia and osteoporosis typically have a smaller waist circumference compared to those with normal BMD values (15). Furthermore, excessive buildup of visceral and subcutaneous fat may negatively impact bone health in women, whether premenopausal or postmenopausal (16).

At present, there is insufficient evidence to establish a relationship between VAI and the prevalence of osteoporosis. The objective of our study was to examine the association of VAI with femur BMD and osteoporosis among the U.S. older adults to fill these knowledge gaps. The secondary goal was to assess the dose–response relationship between VAI and femur BMD at different sites.

## Methods

### Study population

The National Health and Nutrition Examination Survey (NHANES) is a comprehensive investigation designed to assess the health and nutritional status of the non-institutionalized population in the US. It makes use of a multistage, probability-based survey methodology with stratification to ensure accuracy and precision. The National Center for Health Statistics Research Ethics Review Board granted approval for this project, and all participants provided written informed consent prior to their participation (17). According to the related policies, the Institutional Review Board did not require a review for the secondary analysis (18). For this cross-sectional study, we merged the NHANES data from 2005–2010, 2013–2014, and 2017–2020. The participants in our study were all over 60 years of age and had completed interviews and assessments. Participants without complete data regarding BMI, waist circumference (WC), triglycerides (TG), high-density lipoprotein cholesterol (HDL-C), femur BMD, and covariates were excluded from the study.

### Outcome ascertainment

BMD measurements were conducted using dual-energy x-ray absorptiometry scans with Hologic QDR-4500A fan-beam densitometers (Hologic, Inc., Bedford, MA, United States). According to the classification criteria established by the World Health Organization, BMD values in any femur region can be defined as osteoporosis if they fall below −2.5 standard deviations from the reference group for young adults (19). The femoral regions that were evaluated in the study included the total femur, femoral neck, trochanter, and intertrochanter. The corresponding thresholds for osteoporosis were 0.68 g/cm^2^, 0.59 g/cm^2^, 0.49 g/cm^2^, and 0.78 g/cm^2^, respectively (20).

### Exposure measurement

The VAI was used as an exposure variable and was calculated using gender-specific equations, as detailed below. Male: [WC (cm)/39.68 + (1.88 × BMI)] × (TG (mmol/L)/1.03) × (1.31/HDL (mmol/L)); Female: [WC (cm)/36.58 + 1.89 × (BMI)] × (TG (mmol/L)/0.81) × (1.52/HDL (mmol/L)) (21). TG was measured using the Wahlefeld method and HDL was measured using the magnesium sulfate/glucan method (22). The measurements of both TG and HDL were obtained from serum, and participants were required to fast for 9 h. BMI was calculated by dividing the weight in kilograms by the height in meters squared (kg/m^2^). WC was measured using electronic Sports Measurements (Seca Ltd, Medical Scales and Measurement Systems, Birmingham, UK) with an accuracy of millimeters (22). Please refer to the official NHANES website for more detailed information.

### Assessment of other covariates

The choice of variables was determined using previous research (23–25) and clinical observations. NHANES researchers created standardized questionnaires to collect demographic information, including gender, age, race, education level, poverty-to-income ratio (PIR), marital status, smoking status, and work activity. Race was classified as Mexican American, other Hispanic, non-Hispanic white, non-Hispanic black, or other. Education level was categorized as did not graduate from high school, graduated from high school, and college education or above. Marital status was reported as married/living with partner, widowed/divorced/separated, and never married. Smoking status was created from the question: “smoked at least 100 cigarettes in life”. Moderate activity was defined as activity that results in only small elevations in respiration and heart rate, such as brisk walking or carrying lightweight objects for a minimum of 10 min without interruption. The blood urea nitrogen, serum calcium, serum phosphorus, and serum uric acid were measured according to standardized protocols. More information can be obtained from the NHANES website (www.cdc.gov/nchs/nhanes/).

### Statistical analysis

Continuous variables with a normal distribution were reported as mean (standard deviation, [SD]). Alternatively, variables with skewness were reported as median (interquartile range, [IQR]). Categorical variables were presented as frequencies (%). The clinical characteristics of VAI quartiles were compared using one-way ANOVA when the data were normally distributed, Kruskal–Wallis *H* when the distribution was skewed, and the chi-square test for categorical variables analysis. The association between VAI and femur BMD was evaluated using linear regression models (regression coefficients β and 95% confidence interval [CI]). We used logistic regression to investigate the associations between VAI with osteoporosis (odds ratio [OR] and 95% confidence interval [CI]). Both non-adjusted and multivariable adjusted models were applied. In this study, Model 1 was adjusted for gender, age, and race. Model 2 was adjusted for sociodemographic characteristics, including gender, age, race, education level, marital status, PIR, smoking status, and work activity. Model 3 was completely adjusted, including sociodemographic characteristics, blood urea nitrogen, serum calcium, serum phosphorus, and serum uric acid.

In addition, we transformed the VAI into a categorical variable based on the quartile and analyzed the trend *p*-value to validate the outcomes of the VAI as a continuous variable and assess the potential for nonlinearity. To address the nonlinear relationship between the VAI and femur BMD, we utilized a Generalized Additive Model along with smooth curve fitting techniques. Additionally, a two-piecewise linear regression model was employed to further explain the nonlinearity. We further used restricted cubic spline with three knots located at the 5th, 50th, and 95th percentiles of the exposure distribution to assess the dose–response relationship in Model 3. Specifically, when a ratio is represented in a smooth curve, the recursive method can detect inflection points and determine the maximum likelihood model for that point. To ensure the validity and reliability of the results obtained in this study, subgroup analyses were evaluated by multivariable logistic regression models. Potential modifications of the relationship between the VAI and osteoporosis were assessed, including gender, age, race, marital status, and smoking status. The interaction among subgroups was evaluated using the likelihood ratio test. Moreover, two sensitivity analyses were conducted as follows: (1) Participants with extreme VAI outside the mean ± 3 SD were excluded. (2) Participants who had ever taken prednisone or cortisone daily, treated for osteoporosis, had cancer or malignancy, had liver condition, had ever taken estrogen, or had celiac disease were excluded. All analyses were conducted using the statistical software packages R (“rms” R package for conducting restricted cubic spline analysis) and Free Statistics software version 1.7. A two-sided *p*-value <0.05 was regarded as having statistical significance.

## Results

### Study population

**Figure 1:**
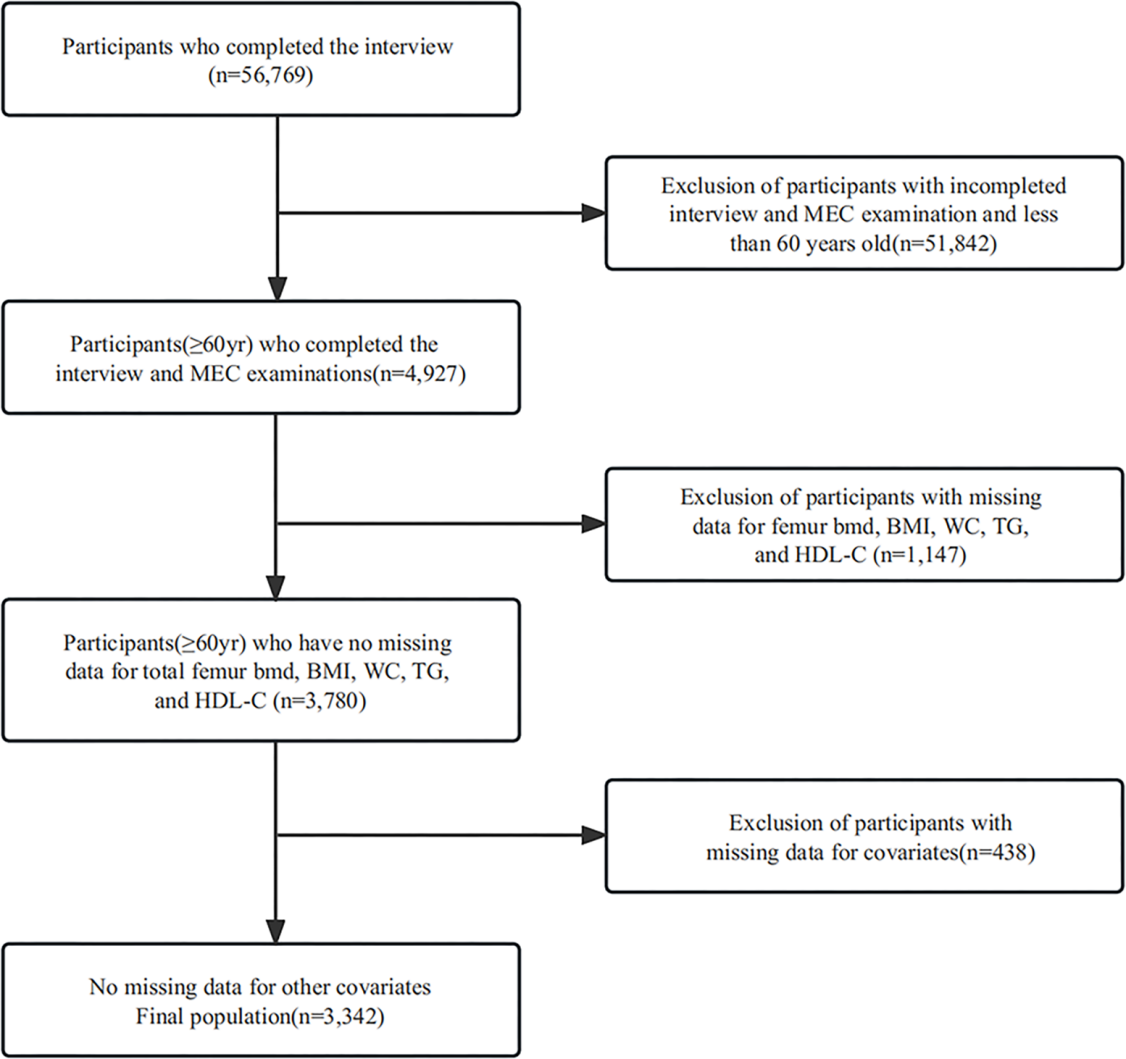
Participants inclusion flowchart.

This study included 56,769 prospective participants from NHANES (2005–2010, 2013–2014, and 2017–2020), of which 4,927 older adults (≥60 years) completed interviews and were subjected to mobile examination center (MEC) screening. Participants with missing data for femur BMD, BMI, WC, TG, and HDL-C (*n* = 1,147) were excluded. Our cross-sectional study included 3,342 participants after the exclusion of those with missing covariate data (*n* = 438) eventually. The study population selection flowchart is shown in **Figure 1**.

### Clinical characteristics

The mean participant age was 69.8 ± 6.9 years, and 1,759 (52.6%) were men. The median VAI was 1.5 (0.9, 2.4). There were 471 (14.1%) participants with osteoporosis. The detailed characteristics of the population by the VAI quartiles are available in **Table 1**. Participants with higher VAI often tended to be younger, female, had a lower educational level and family income, smoked more cigarettes, had less work activity, and had higher serum phosphorus and serum uric acid level.

**Table 1:** Clinical characteristics of the study population.

Variables	Visceral adiposity index					*p*-value
	Total (*n* = 3341)	Q1 (*n* = 825)	Q2 (*n* = 836)	Q3 (*n* = 844)	Q4 (*n* = 836)	
Gender, *n* (%)						<0.001
Male	1,759 (52.6)	489 (59.3)	466 (55.7)	414 (49.1)	390 (46.7)	
Female	1,582 (47.4)	336 (40.7)	370 (44.3)	430 (50.9)	446 (53.3)	
Age, (years)	69.8 ± 6.9	70.0 ± 7.0	69.8 ± 6.9	70.0 ± 6.8	69.3 ± 6.9	0.133
Race, *n* (%)						
Mexican American	386 (11.6)	53 (6.4)	89 (10.6)	110 (13)	134 (16)	<0.001
Other Hispanic	277 (8.3)	48 (5.8)	79 (9.4)	70 (8.3)	80 (9.6)	
Non-Hispanic White	1,798 (53.8)	410 (49.7)	416 (49.8)	475 (56.3)	497 (59.4)	
Non-Hispanic Black	637 (19.1)	241 (29.2)	196 (23.4)	126 (14.9)	74 (8.9)	
Other Race	243 (7.3)	73 (8.8)	56 (6.7)	63 (7.5)	51 (6.1)	
Education level, *n* (%)						<0.001
Did not graduate from high school	936 (28.0)	181 (21.9)	229 (27.4)	237 (28.1)	289 (34.6)	
Graduated from high school	837 (25.1)	188 (22.8)	210 (25.1)	226 (26.8)	213 (25.5)	
College education or above	1,568 (46.9)	456 (55.3)	397 (47.5)	381 (45.1)	334 (40)	
Marital status, *n* (%)						0.643
Married/Living with Partner	2,060 (61.7)	517 (62.7)	520 (62.2)	516 (61.1)	507 (60.6)	
Widowed/Divorced/Separated	1,138 (34.1)	265 (32.1)	282 (33.7)	294 (34.8)	297 (35.5)	
Never married	143 (4.3)	43 (5.2)	34 (4.1)	34 (4)	32 (3.8)	
PIR	2.3 (1.3, 4.2)	2.7 (1.5, 5.0)	2.3 (1.3, 4.6)	2.2 (1.4, 3.8)	2.0 (1.1, 3.6)	<0.001
Smoking status, *n* (%)						0.062
Smoked at least 100 cigarettes	1,743 (52.2)	417 (50.5)	418 (50)	440 (52.1)	468 (56)	
Work activity, *n* (%)						0.356
Moderate activity	1,209 (36.2)	319 (38.7)	296 (35.4)	304 (36)	290 (34.7)	
Blood urea nitrogen (mg/dL)	16.4 ± 7.0	16.1 ± 6.3	16.2 ± 6.7	16.2 ± 6.6	16.9 ± 8.0	0.123
Serum calcium (mg/dL)	9.4 ± 0.4	9.4 ± 0.4	9.4 ± 0.4	9.4 ± 0.4	9.4 ± 0.4	0.434
Serum phosphorus (mg/dL)	3.6 ± 0.5	3.6 ± 0.5	3.6 ± 0.6	3.6 ± 0.5	3.7 ± 0.5	0.013
Serum uric acid (mg/dL)	5.8 ± 1.4	5.4 ± 1.3	5.7 ± 1.4	5.9 ± 1.4	6.1 ± 1.5	<0.001
Total femur BMD (g/cm^2^)	0.9 ± 0.2	0.9 ± 0.2	0.9 ± 0.2	0.9 ± 0.2	0.9 ± 0.2	0.058
Femur neck BMD (g/cm^2^)	0.8 ± 0.1	0.8 ± 0.1	0.8 ± 0.1	0.7 ± 0.1	0.8 ± 0.1	0.447
Trochanter BMD (g/cm^2^)	0.7 ± 0.1	0.7 ± 0.1	0.7 ± 0.1	0.7 ± 0.1	0.7 ± 0.1	0.183
Intertrochanter BMD (g/cm^2^)	1.1 ± 0.2	1.1 ± 0.2	1.1 ± 0.2	1.1 ± 0.2	1.1 ± 0.2	0.021
Osteoporosis, *n* (%)	471 (14.1)	119 (14.4)	115 (13.8)	128 (15.2)	109 (13)	0.632
Visceral adiposity index	1.5 (0.9, 2.4)	0.7 (0.5, 0.8)	1.1 (1.0, 1.3)	1.8 (1.6, 2.1)	3.3 (2.8, 4.4)	<0.001

Data were mean ± SD or median (IQR) for skewed variables or numbers (proportions) for categorical variables.

PIR, ratio of family income to poverty; BMD, bone mineral density.

### Associations of the VAI with femur BMD and osteoporosis

The univariate analysis demonstrated that gender, age, education level, marital status, PIR, smoking status, work activity, blood urea nitrogen, serum phosphorus, and serum uric acid were associated with osteoporosis (**Supplementary Table S1**).

After adjustment in multivariable analyses, the VAI was significantly associated with femur BMD and osteoporosis. When considering the VAI as a continuous variable, the adjusted OR for osteoporosis was 0.88 (95% CI: 0.81–0.96, *p* = 0.002) in the fully adjusted model. Compared with participants with the lowest VAI in the 1st Quartile (≤0.92), the adjusted OR value for the VAI and osteoporosis in the 4th Quartile (≥2.39) was 0.61 (95% CI: 0.44–0.85, *p* = 0.003) in Model 3 (**Table 2**). When the VAI was assessed as a continuous variable, the regression coefficients β were 0.006 (95% CI: 0.004–0.009, *p* < 0.001), 0.004 (95% CI: 0.002–0.006, *p* = 0.001), 0.005 (95% CI: 0.003–0.007, *p* < 0.001), and 0.007 (95% CI: 0.004–0.010, *p* < 0.001) for total femur BMD, femur neck BMD, trochanter BMD, and intertrochanter BMD in the fully adjusted model (Model 3), respectively. After adjusting for all variables, analysis using quartiles revealed a significant positive correlation between VAI and femur BMD (**Table 3**). All of the models were statistically significant (**Tables 2**, **3**, *p* for trend<0.001).

**Table 2:** Association between visceral adiposity index and osteoporosis in the multiple regression model.

Variable	Unadjusted		Model 1		Model 2		Model 3	
	OR (95% CI)	P-value	OR (95% CI)	P-value	OR (95% CI)	P-value	OR (95% CI)	P-value
Visceral adiposity index	0.95 (0.89–1.01)	0.119	0.89 (0.83–0.97)	0.005	0.85 (0.79–0.93)	<0.001	0.88 (0.81–0.96)	0.002
1st Quartile (≤0.92)	1 (Ref)		1 (Ref)		1 (Ref)		1 (Ref)	
2st Quartile (0.93-1.45)	0.95 (0.72–1.25)	0.696	0.87 (0.64–1.17)	0.351	0.80 (0.59–1.08)	0.144	0.85 (0.62–1.15)	0.290
3st Quartile (1.46-2.38)	1.06 (0.81–1.39)	0.670	0.80 (0.59–1.08)	0.140	0.72 (0.53–0.98)	0.034	0.80 (0.59–1.09)	0.164
4st Quartile (≥2.39)	0.89 (0.67–1.18)	0.412	0.64 (0.47–0.88)	0.005	0.53 (0.39–0.73)	<0.001	0.61 (0.44–0.85)	0.003
*p* for trend		<0.001		<0.001		<0.001		<0.001

Model 1 adjusted for gender, age, and race.

Model 2 adjusted for gender, age, race, education level, marital status, PIR, smoking status, and work activity.

Model 3 adjusted for gender, age, race, education level, marital status, PIR, smoking status, work activity, blood urea nitrogen, serum calcium, serum phosphorus, and serum uric acid.

Ref, reference; PIR, ratio of family income to poverty; BMD, bone mineral density; VAI, visceral adiposity index.

**Table 3:** Association between visceral adiposity index and femur BMD in the multiple regression model.

Variable	Unadjusted		Model 1		Model 2		Model 3	
	β (95% CI)	P-value	β (95% CI)	P-value	β (95% CI)	P-value	β (95% CI)	P-value
Total femur BMD (g/cm^2^)								
Visceral adiposity index	0.004 (0.001–0.007)	0.003	0.006 (0.004–0.009)	<0.001	0.008 (0.005–0.010)	<0.001	0.006 (0.004–0.009)	<0.001
1st Quartile (≤0.92)	0 (Ref)		0 (Ref)		0 (Ref)		0 (Ref)	
2st Quartile (0.93–1.45)	0.013 (−0.004–0.029)	0.128	0.021 (0.008–0.034)	0.002	0.023 (0.009–0.037)	<0.001	0.019 (0.006–0.032)	0.005
3st Quartile (1.46–2.38)	0.008 (−0.008–0.024)	0.344	0.035 (0.021–0.048)	<0.001	0.039 (0.026–0.053)	<0.001	0.031 (0.018–0.045)	<0.001
4st Quartile (≥2.39)	0.022 (0.006–0.038)	0.008	0.053 (0.040–0.067)	<0.001	0.062 (0.048–0.075)	<0.001	0.051 (0.037–0.065)	<0.001
*p* for trend		<0.001		<0.001		<0.001		<0.001
Femur neck BMD (g/cm^2^)								
Visceral adiposity index	0.002 (−0.001–0.005)	0.131	0.004 (0.002–0.006)	<0.001	0.005 (0.003–0.007)	<0.001	0.004 (0.002–0.006)	0.001
1st Quartile (≤0.92)	0 (Ref)		0 (Ref)		0 (Ref)		0 (Ref)	
2st Quartile (0.93–1.45)	0.005 (−0.009–0.019)	0.491	0.012 (0.000–0.025)	0.047	0.014 (0.002–0.027)	0.022	0.011 (−0.001–0.023)	0.083
3st Quartile (1.46–2.38)	−0.001 (−0.015–0.013)	0.855	0.022 (0.010–0.034)	<0.001	0.026 (0.013–0.038)	<0.001	0.019 (0.007–0.032)	0.002
4st Quartile (≥2.39)	0.009 (−0.005–0.023)	0.206	0.037 (0.024–0.049)	<0.001	0.043 (0.03–0.056)	<0.001	0.035 (0.022–0.048)	<0.001
*p* for trend		<0.001		<0.001		<0.001		<0.001
Trochanter BMD (g/cm^2^)								
Visceral adiposity index	0.003 (0.001–0.006)	0.014	0.005 (0.003–0.007)	0.001	0.006 (0.004–0.008)	<0.001	0.005 (0.003–0.007)	<0.001
1st Quartile (≤0.92)	0 (Ref)		0 (Ref)		0 (Ref)		0 (Ref)	
2st Quartile (0.93–1.45)	0.012 (−0.002–0.026)	0.092	0.019 (0.007–0.031)	0.002	0.021 (0.010–0.033)	<0.001	0.017 (0.006–0.029)	0.004
3st Quartile (1.46–2.38)	0.008 (−0.006–0.022)	0.256	0.029 (0.017–0.041)	<0.001	0.033 (0.022–0.045)	<0.001	0.027 (0.015–0.039)	<0.001
4st Quartile (≥2.39)	0.015 (0.001–0.028)	0.039	0.039 (0.027–0.052)	<0.001	0.047 (0.035–0.059)	<0.001	0.038 (0.026–0.051)	<0.001
*p* for trend		<0.001		<0.001		<0.001		<0.001
Intertrochanter BMD (g/cm^2^)								
Visceral adiposity index	0.006 (0.002–0.009)	0.002	0.008 (0.005–0.011)	<0.001	0.009 (0.006–0.012)	<0.001	0.007 (0.004–0.010)	<0.001
1st Quartile (≤0.92)	0 (Ref)		0 (Ref)		0 (Ref)		0 (Ref)	
2st Quartile (0.93–1.45)	0.014 (−0.005–0.033)	0.141	0.023 (0.007–0.039)	0.004	0.026 (0.01–0.042)	0.001	0.021 (0.005–0.037)	0.010
3st Quartile (1.46–2.38)	0.011 (−0.008–0.030)	0.263	0.041 (0.025–0.057)	<0.001	0.046 (0.03–0.062)	<0.001	0.037 (0.020–0.053)	<0.001
4st Quartile (≥2.39)	0.030 (0.011–0.049)	0.002	0.065 (0.049–0.081)	<0.001	0.074 (0.058–0.091)	<0.001	0.061 (0.044–0.078)	<0.001
*p* for trend		<0.001		<0.001		<0.001		<0.001

Model 1 adjusted for gender, age, and race.

Model 2 adjusted for gender, age, race, education level, marital status, PIR, smoking status, and work activity.

Model 3 adjusted for gender, age, race, education level, marital status, PIR, smoking status, work activity, blood urea nitrogen, serum calcium, serum phosphorus, and serum uric acid.

Ref, reference; PIR, ratio of family income to poverty; BMD, bone mineral density; VAI, visceral adiposity index.

**Figure 2:**
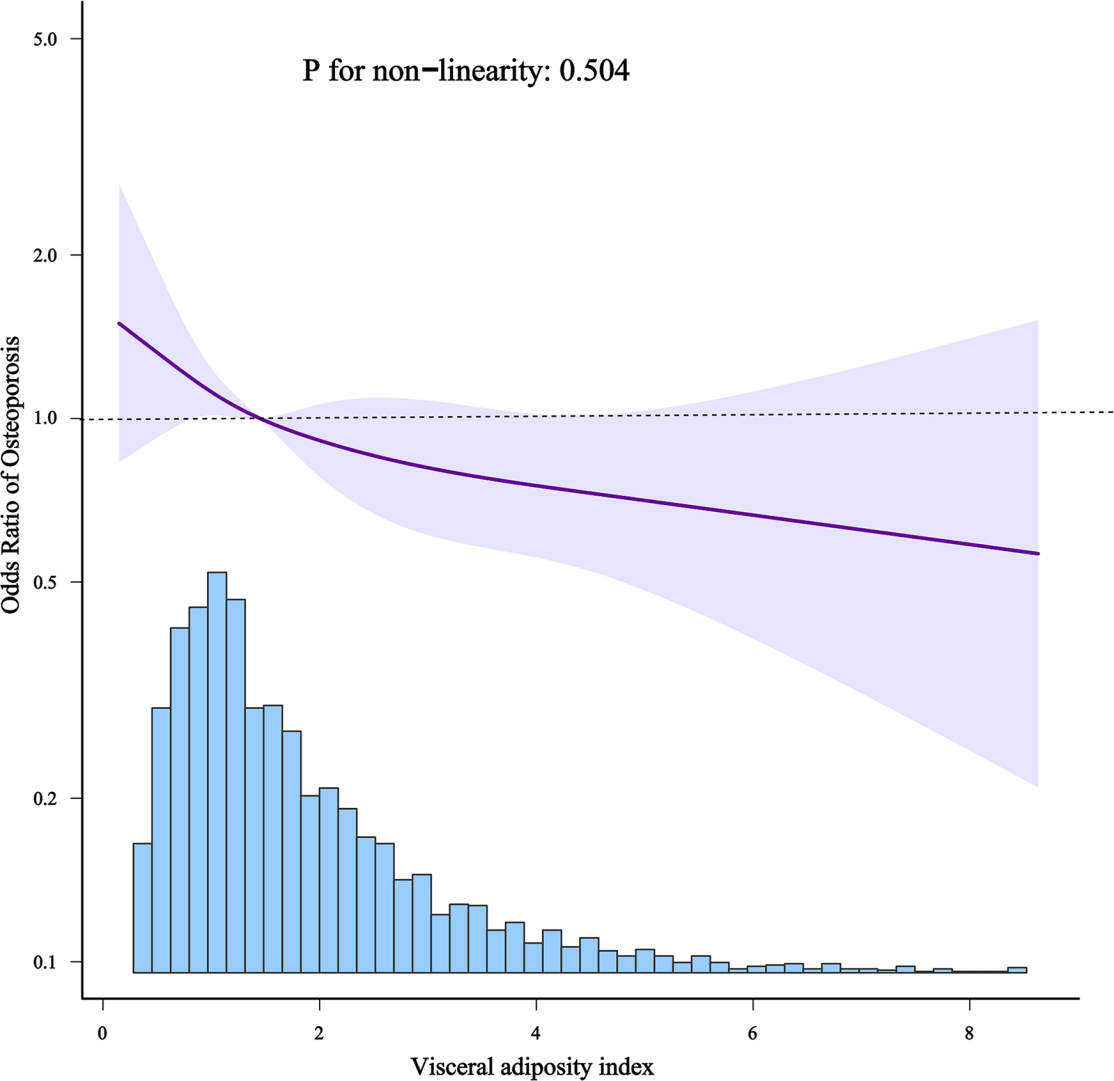
Restricted cubic spline model of the odds ratios of VAI and osteoporosis.

**Figure 3:**
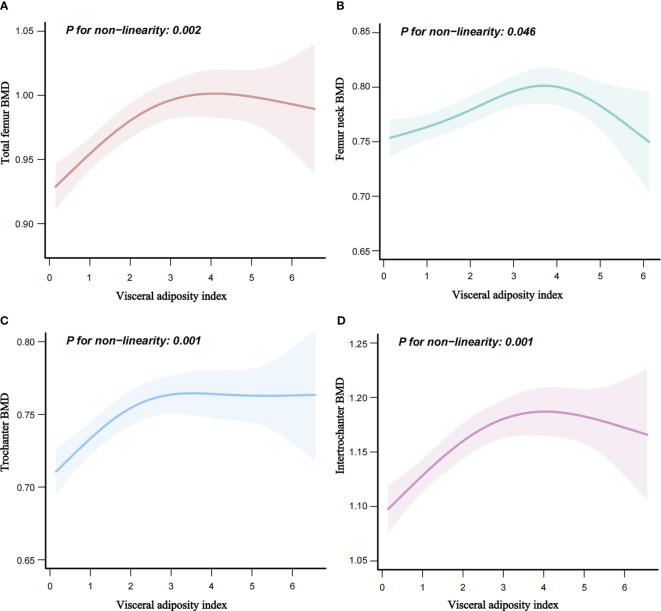
Nonlinear relationship of VAI and femur BMD. **(A)** Nonlinear relationship of VAI and total femur BMD. **(B)** Nonlinear relationship of VAI and femur neck BMD. **(C)** Nonlinear relationship of VAI and trochanter BMD. **(D)** Nonlinear relationship of VAI and intertrochanter BMD. BMD, bone mineral density (g/cm^2^).

There was a linear relationship between VAI and osteoporosis (**Figure 2**). Additionally, an inverted L-shaped curve was observed in the correlation between VAI and BMD in different areas of the femur, including total femur, trochanter, and intertrochanter regions (nonlinear, *p* = 0.002, *p* = 0.001, *p* = 0.001, respectively) after adjusting for all covariates. Interestingly, the relationship between the VAI and femur neck BMD exhibited an inverted U-shaped curve (**Figure 3**). Femur neck BMD was positively correlated with the VAI until it peaked at 3.9 (β = 0.014 [95% CI: 0.009–0.020]). However, when the VAI was higher than 3.9, the femur neck BMD decreased significantly (β = −0.044 [95% CI: −0.075 to −0.013]) (**Table 4**). The solid line represents the estimated risk, and the light-shaded area represents a point-wise 95% confidence interval adjusted for Model 3 (**Figures 2**, **3**).

**Table 4:** Threshold effect analysis of the relationship of visceral adiposity index with femur BMD.

	Visceral adiposity index	Adjusted model	
**Total femur BMD (g/cm^2^)**		**β (95% CI)**	***p*-value**
	<3.0	0.022 (0.014–0.030)	<0.001
	≥3.0	−0.009 (−0.024–0.005)	0.195
	Likelihood ratio test		<0.001
**Femur neck BMD (g/cm^2^)**	<3.9	0.014 (0.009–0.020)	<0.001
	≥3.9	−0.044 (−0.075–0.013)	0.006
	Likelihood ratio test		<0.001
**Trochanter BMD (g/cm^2^)**	<2.4	0.023 (0.014–0.032)	<0.001
	≥2.4	0.002 (−0.006–0.011)	0.582
	Likelihood ratio test		<0.001
**Intertrochanter BMD (g/cm^2^)**	<3.2	0.031 (0.022–0.039)	<0.001
	≥3.2	−0.007 (−0.025–0.011)	0.420
	Likelihood ratio test		<0.001

Adjusted for Model 3 (gender, age, race, education level, marital status, PIR, smoking status, work activity, blood urea nitrogen, serum calcium, serum phosphorus, and serum uric acid).

### Stratified analyses based on additional variables

**Figure 4:**
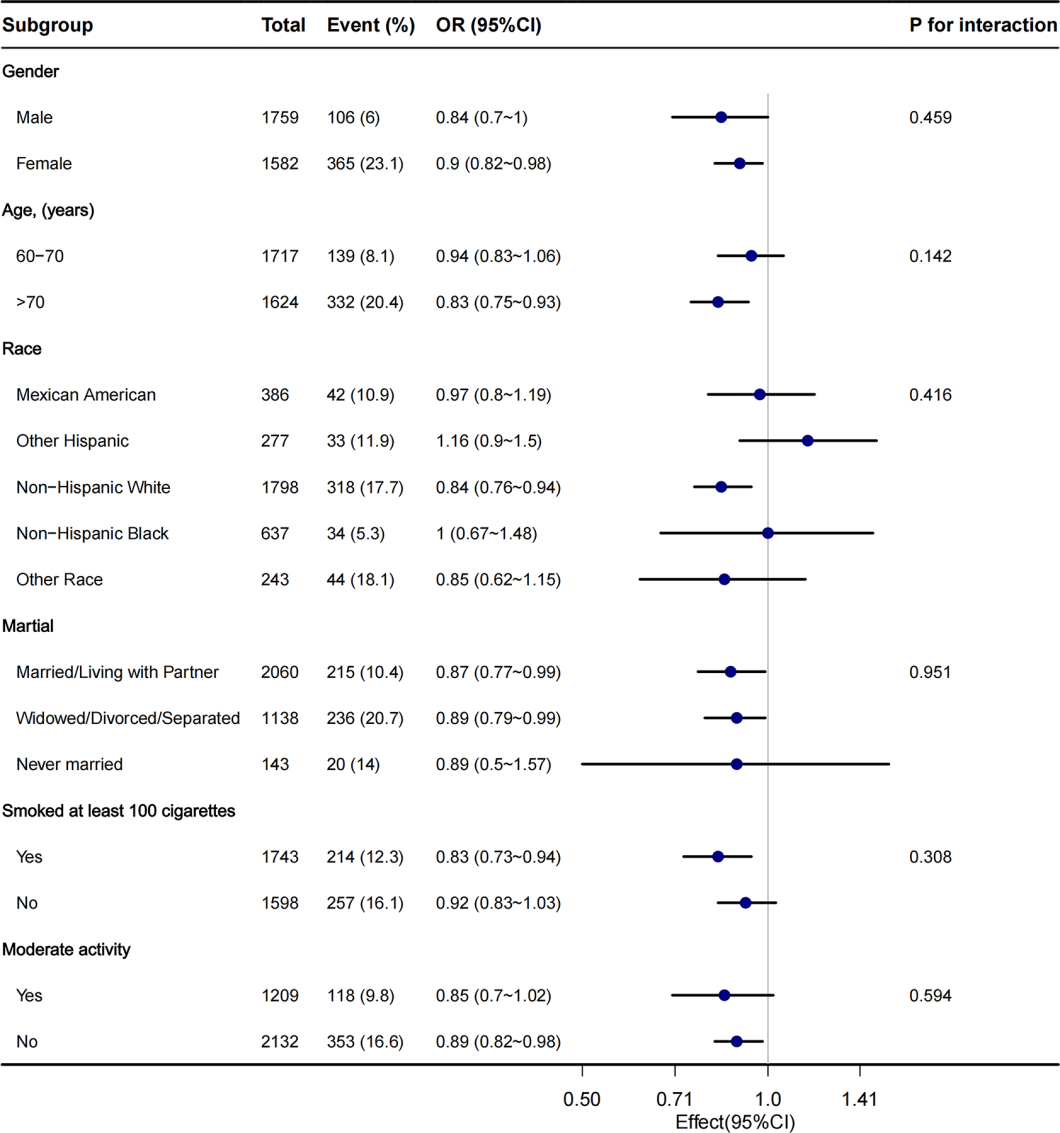
The relationship between VAI and osteoporosis according to basic features.

Stratified analyses were performed in several subgroups to assess the potential effect modification of the relationship between VAI and osteoporosis. Significant interactions were not observed in any of the subgroups after stratifying by gender, age, race, marital status, and smoking status (**Figure 4**).

### Sensitivity analysis

A total of 3,296 participants were left after excluding the participants with extreme VAI (outside the mean ± 3 SD were excluded), and the association between VAI and osteoporosis remained stable. When considering the VAI as a continuous variable, the adjusted OR for osteoporosis was 0.88 (95% CI: 0.80–0.96, *p* = 0.007) in the fully adjusted model. Compared with participants with the lowest VAI in the 1st Quartile (≤0.92), the adjusted OR value for the VAI and osteoporosis in 4th Quartile (≥2.39) was 0.63 (95% CI: 0.46–0.88, *p* = 0.007) (**Supplementary Table S2**). After excluding participants who had ever taken prednisone or cortisone daily, been treated for osteoporosis, had cancer or malignancy, had liver conditions, ever taken estrogen, or had celiac disease, 2,246 participants remained, and the association between VAI and osteoporosis remained consistent. When considering the VAI as a continuous variable, the adjusted OR for osteoporosis was 0.86 (95% CI: 0.77–0.96, *p* = 0.007) in the fully adjusted model. Compared with participants with the lowest VAI in the 1st Quartile (≤0.92), the adjusted OR value for the VAI and osteoporosis in 4th Quartile (≥2.40) was 0.52 (95% CI: 0.33–0.81, *p* = 0.004) (**Supplementary Table S3**).

## Discussion

In this large population-based cross-sectional study of the older adults in the United States using 2005–2020 NHANES data, the results showed that VAI, whether as a continuous or categorical variable, was positively and linearly associated with osteoporosis when adjusted for potential confounding factors. We further revealed a nonlinear relationship between VAI and femur BMD. Specifically, an inverted L-shaped correlation was found between VAI and BMD across some regions of the femur such as the total femur, trochanter, and intertrochanter areas, with an inflection point of almost 3.0, 2.4, and 3.2, respectively. What is of interest is that the relationship between VAI and femur neck BMD displayed a curvilinear pattern resembling an inverted U-shape. The femur neck BMD was significantly reduced with the excessive VAI values. The most beneficial VAI value for femur neck BMD is 3.9. As far as we know, there are no other studies investigating the association of the VAI with femur BMD and osteoporosis among the U.S. older adults.

BMD typically showed higher values in individuals with greater weight or BMI (26, 27). Furthermore, a study has indicated that total body fat emerges as the most significant predictor of BMD throughout the entire skeletal structure (28), while a lower amount of body fat is identified as an independent risk factor contributing to greater lumbar spine bone loss in women (29). The research also indicated that obesity significantly reduces the risk of osteoporosis, osteopenia, and low bone mass in all participants (30). One reasonable mechanism explaining the increase in BMD in obese individuals is associated with the increased mechanical load and strain related to obesity (8). As body fat increases in obese individuals, it not only leads to passive loading but also increases muscle strain, which has a favorable impact on bone geometry and modeling (31). In fact, increased body fat from moderate obesity can have beneficial effects on bone health by promoting skeletal plasticity and modeling, which assists in maintaining bone mass and reducing bone loss, especially in the older adults (8). This has significant advantages in preventing fragility fractures. However, severe obesity, in particular, may be associated with an elevated risk of fractures (32, 33). Studies have indicated that women with abdominal obesity experience lower concentrations of serum dihydrotestosterone (DHT) (34), and that DHT has a significant inhibitory effect on estrogen synthesis *in vivo* (35). In low DHT environments, estrogen accumulation can play a role in maintaining optimal bone health. In contrast, moderate visceral fat accumulation in men is significantly positively correlated with endogenous adrenal steroid levels (36). Endogenous adrenal steroids have a noteworthy contribution to elevated estrogen levels, which could be why elevated VAI may be associated with reducing the risk of osteoporosis (37). In a cohort study involving postmenopausal women in Turkey, it was discovered that after controlling for potential confounding risks, higher VAI may contribute to a reduced risk of osteoporosis (38). This study supports our results.

In addition, we discovered an inverted L-shaped and inverted U-shaped relationship between VAI and femur BMD at different sites. The beneficial effect of increasing the level of VAI on femur BMD seemed to peak in those with considerably high VAI. Individuals with extremely high VAI levels tend to experience a decrease in femur BMD, which may indicate a potential negative impact on bone health. Several published research findings suggest a possible association between more visceral adipose tissue and reduced BMD, indicating that excessive VAI may elevate the risk of osteoporosis. When body fat percentage is below 33%, BMD is positively correlated with body fat content, which can decrease the risk of fragility fracture. However, when the body fat percentage exceeds 33%, body fat content in most skeletal sites is negatively related to BMD, which increases the risk of osteoporosis and fragility fracture (39–42). Activation of the Wnt/β-catenin signaling pathway has been shown to effectively inhibit the proliferation and differentiation of adipocytes, leading to reduced accumulation of visceral fat (43). The positive establishment and activation of the Wnt/β-catenin signaling pathway is crucial for the value-added of osteoblasts, and mutations in the LRP5 gene within this pathway have been identified as an important cause of osteoporosis (44). Therefore, it is reasonable to suspect that excessive obesity or a high VAI may impact the development of osteoporosis by disrupting the Wnt/β-catenin signaling pathway. High levels of VAI are an independent predictor of the development of type 2 diabetes (45), which is an important risk factor for osteoporosis (46, 47). Abnormally high levels of visceral and ectopic fat accumulation can lead to massive infiltration of local environmental macrophages and induce the release of inflammatory factors such as IL-6. These factors have been shown to cause reduced bone content and enhanced bone resorption, ultimately inducing the development of osteoporosis (48). The visceral adipose tissue derived from CT scans has demonstrated a statistically significant correlation with decreased bone and muscle density, even after adjusting for age, gender, and BMI (49). Another study has already demonstrated that visceral adipose tissue has an adverse effect on BMD in premenopausal obese women and has proposed that this association may be mediated by IGF-1 (50). Visceral adipose tissue has been shown to be negatively associated with BMD in a study based on the NHANES database; however, this study did not include older adults (45).

The strengths of our study included adjusting for multiple biochemical indicators that were closely associated with osteoporosis (24, 25, 51) and the relatively large sample size. This study also has some limitations. Firstly, femur BMD data were not collected in NHANES 2011–2012 and 2015–2016. Therefore, we were unable to further validate the results with more cycles of data. Secondly, owing to the inherent limitations of cross-sectional studies, the causal association of the VAI with femur BMD and osteoporosis cannot be determined, and current relationships may be bidirectional. Thirdly, lumbar spine BMD was not included in this study mainly because degenerative changes in the lumbar spine are more pronounced in older adults, which can lead to falsely elevated measurements (52, 53). There are more missing values for lumbar spine bone density. However, there are guidelines that consider the lumbar spine to be the preferred site for efficacy assessment (54). This may have an impact on the results. Fourthly, an elevated BMD does not necessarily reflect superior bone quality. A more accurate assessment of bone quality could be obtained through HR-pQCT. However, this was not within the scope of our study. Lastly, despite adjusting for potential confounding factors and conducting sensitivity analyses, because of the limitations of the NHANES database, we were unable to access information regarding patients’ dietary patterns, specific dietary characteristics, and other potential confounders such as whether participants had diseases associated with absorption disorders, underwent abdominal surgeries leading to absorption issues, or had active infectious foci, which could still potentially impact the results. More research is needed to supplement our understanding of the bidirectional modulating effects of obesity on BMD and skeletal remodeling, as well as the impact threshold. Further high-quality prospective studies are required to clarify the potential mechanisms linking VAI and osteoporosis.

## Conclusion

In this cross-sectional study based on the NHANES from 2005 to 2020, we found that an increased VAI is independently linked to a higher prevalence of osteoporosis among the U.S. older adults. Interestingly, our results also revealed a nonlinear relationship between VAI and femur BMD at various sites, characterized by an inverted L-shape and an inverted U-shape, suggesting that once VAI reaches a certain threshold, femur BMD no longer increases and may even decrease. This implies that a moderate buildup of visceral fat might have a positive impact on bone health, whereas an excessive amount of visceral fat could potentially lead to adverse effects. VAI could serve as a valuable indicator for clinically assessing the skeletal health of the older adults.

## Data availability statement

The original contributions presented in the study are included in the article/**Supplementary Material**. Further inquiries can be directed to the corresponding author.

## Ethics statement

The studies involving humans were approved by National Center for Health Statistics research ethics review board. The studies were conducted in accordance with the local legislation and institutional requirements. The human samples used in this study were acquired from gifted from another research group. Written informed consent for participation was not required from the participants or the participants’ legal guardians/next of kin in accordance with the national legislation and institutional requirements.

## Author contributions

Conceptualization, ZL, AS, and JH. Data curation, AS and SW. Formal analysis, AS. Funding acquisition, ZL. Methodology, AS. Writing—original draft, AS, JH, SW, and FY. Writing—review and editing, ZL, AS, and JH. All authors have read and agreed to the published version of the manuscript.
