# Supplementary material for: Association of the visceral adiposity index with femur bone mineral density and osteoporosis among the U.S. older adults from NHANES 2005–2020: a cross-sectional study
Source: Front Endocrinol (Lausanne). 2023 Nov 2;14:1231527. doi: 10.3389/fendo.2023.1231527 (PMC10653335; doi:10.3389/fendo.2023.1231527)
Supplement: Supplementary file 1 [file Table_1.doc]

**Table S1.** Results of univariate analysis of osteoporosis.

| **Variable** | **OR(95%CI)** | **P-value** |
| --- | --- | --- |
| Gender, n (%) |  |  |
| Male | Ref |  |
| Female | 4.68 (3.72~5.88) | <0.001 |
| Age, (years) | 1.1 (1.08~1.11) | <0.001 |
| Race, n (%) |  |  |
| Mexican American | Ref |  |
| Other Hispanic | 1.11 (0.68~1.8) | 0.679 |
| Non-Hispanic White | 1.76 (1.25~2.48) | 0.001 |
| Non-Hispanic Black | 0.46 (0.29~0.74) | 0.001 |
| Other Race | 1.81 (1.15~2.86) | 0.011 |
| Education level, n (%) |  |  |
| Did not graduate from high school | Ref |  |
| Graduated from high school | 0.95 (0.74~1.23) | 0.697 |
| College education or above | 0.68 (0.54~0.86) | 0.001 |
| Marital status, n (%) |  |  |
| Married/Living with Partner | Ref |  |
| Widowed/Divorced/Separated | 2.25 (1.84~2.75) | <0.001 |
| Never married | 1.4 (0.85~2.28) | 0.186 |
| PIR | 0.84 (0.79~0.9) | <0.001 |
| Smoking status, n (%) |  |  |
| Smoked at least 100 cigarettes | 1.37 (1.13~1.67) | 0.002 |
| Work activity, n (%) |  |  |
| Moderate activity | 1.83 (1.47~2.29) | <0.001 |
| Blood urea nitrogen, (mg/dL) | 1.02 (1~1.03) | 0.013 |
| Serum calcium, (mg/dL) | 0.97 (0.74~1.26) | 0.805 |
| Serum phosphorus, (mg/dL) | 1.85 (1.55~2.22) | <0.001 |
| Serum uric acid, (mg/dL) | 0.78 (0.73~0.84) | <0.001 |

Ref, reference; PIR, ratio of family income to poverty; BMD, bone mineral density.
