# Supplementary material for: Association of the visceral adiposity index with femur bone mineral density and osteoporosis among the U.S. older adults from NHANES 2005–2020: a cross-sectional study
Source: Front Endocrinol (Lausanne). 2023 Nov 2;14:1231527. doi: 10.3389/fendo.2023.1231527 (PMC10653335; doi:10.3389/fendo.2023.1231527)
Supplement: Supplementary file 2 [file Table_2.doc]

**Table S2.** Association between visceral adiposity index and osteoporosis in multiple regression model. Extreme VAI was not included.

| **Variable** | **Unadjusted** | |  | **Model 1** | |  | **Model 2** | |  | **Model 3** | |
| --- | --- | --- | --- | --- | --- | --- | --- | --- | --- | --- | --- |
|  | **OR (95%CI)** | **P-value** |  | **OR (95%CI)** | **P-value** |  | **OR (95%CI)** | **P-value** |  | **OR (95%CI)** | **P-value** |
| Visceral adiposity index | 0.97 (0.9~1.05) | 0.519 |  | 0.89 (0.81~0.97) | 0.01 |  | 0.84 (0.77~0.93) | <0.001 |  | 0.88 (0.8~0.96) | 0.007 |
| 1st Quartile (≤0.92) | 1 (Ref) |  |  | 1 (Ref) |  |  | 1 (Ref) |  |  | 1 (Ref) |  |
| 2st Quartile (0.91-1.45) | 0.93 (0.71~1.23) | 0.626 |  | 0.85 (0.63~1.16) | 0.311 |  | 0.79 (0.58~1.07) | 0.129 |  | 0.83 (0.61~1.14) | 0.253 |
| 3st Quartile (1.46-2.38) | 1.1 (0.84~1.45) | 0.493 |  | 0.83 (0.61~1.12) | 0.218 |  | 0.75 (0.55~1.02) | 0.063 |  | 0.83 (0.61~1.14) | 0.259 |
| 4st Quartile (≥2.39) | 0.92 (0.7~1.22) | 0.566 |  | 0.66 (0.48~0.9) | 0.009 |  | 0.55 (0.4~0.76) | <0.001 |  | 0.63 (0.46~0.88) | 0.007 |
| P for trend |  | <0.001 |  |  | <0.001 |  |  | <0.001 |  |  | <0.001 |

Model 1 adjust for Gender, Age, Race.

Model 2 adjust for Model 1+Education level, Marital status, PIR, Smoking status, Work activity.

Model 3 adjust for Model 1+Blood urea nitrogen, Serum calcium, Serum phosphorus, Serum uric acid.

Ref, reference; PIR, ratio of family income to poverty; BMD, bone mineral density; VAI, visceral adiposity index.
