# Supplementary material for: Association of the visceral adiposity index with femur bone mineral density and osteoporosis among the U.S. older adults from NHANES 2005–2020: a cross-sectional study
Source: Front Endocrinol (Lausanne). 2023 Nov 2;14:1231527. doi: 10.3389/fendo.2023.1231527 (PMC10653335; doi:10.3389/fendo.2023.1231527)
Supplement: Supplementary file 3 [file Table_3.doc]

**Table S3.** Association between visceral adiposity index and osteoporosis in multiple regression model.

| **Variable** | **Unadjusted** | |  | **Model 1** | |  | **Model 2** | |  | **Model 3** | |
| --- | --- | --- | --- | --- | --- | --- | --- | --- | --- | --- | --- |
|  | **OR (95%CI)** | **P-value** |  | **OR (95%CI)** | **P-value** |  | **OR (95%CI)** | **P-value** |  | **OR (95%CI)** | **P-value** |
| Visceral adiposity index | 0.95 (0.88~1.04) | 0.279 |  | 0.88 (0.79~0.98) | 0.015 |  | 0.84 (0.75~0.94) | 0.002 |  | 0.86 (0.77~0.96) | 0.007 |
| 1st Quartile (≤0.92) | 1 (Ref) |  |  | 1 (Ref) |  |  | 1 (Ref) |  |  | 1 (Ref) |  |
| 2st Quartile (0.91-1.44) | 0.92 (0.64~1.33) | 0.66 |  | 0.8 (0.54~1.2) | 0.287 |  | 0.75 (0.5~1.13) | 0.176 |  | 0.8 (0.53~1.21) | 0.284 |
| 3st Quartile (1.45-2.39) | 1.12 (0.79~1.6) | 0.529 |  | 0.82 (0.55~1.21) | 0.314 |  | 0.76 (0.51~1.12) | 0.167 |  | 0.86 (0.57~1.29) | 0.461 |
| 4st Quartile (≥2.40) | 0.82 (0.56~1.19) | 0.297 |  | 0.55 (0.36~0.83) | 0.005 |  | 0.46 (0.3~0.7) | <0.001 |  | 0.52 (0.33~0.81) | 0.004 |
| P for trend |  | <0.001 |  |  | <0.001 |  |  | <0.001 |  |  | <0.001 |

Model 1 adjust for Gender, Age, Race.

Model 2 adjust for Model 1+Education level, Marital status, PIR, Smoking status, Work activity.

Model 3 adjust for Model 1+Blood urea nitrogen, Serum calcium, Serum phosphorus, Serum uric acid.

Ref, reference; PIR, ratio of family income to poverty; BMD, bone mineral density; VAI, visceral adiposity index.
